# HLA-C and KIR permutations influence chronic obstructive pulmonary disease risk

**DOI:** 10.1172/jci.insight.150187

**Published:** 2021-10-08

**Authors:** Takudzwa Mkorombindo, Thi K. Tran-Nguyen, Kaiyu Yuan, Yingze Zhang, Jianmin Xue, Gerard J. Criner, Young-il Kim, Joseph M. Pilewski, Amit Gaggar, Michael H. Cho, Frank C. Sciurba, Steven R. Duncan

**Affiliations:** 1 Division of Pulmonary, Allergy & Critical Care Medicine, Department of Medicine, University of Alabama at Birmingham, Birmingham, Alabama, USA.; 2 Division of Pulmonary, Allergy and Critical Care Medicine, Department of Medicine, University of Pittsburgh, Pittsburgh, Pennsylvania, USA.; 3 Department of Thoracic Medicine and Surgery, Lewis Katz School of Medicine, Temple University, Philadelphia, Pennsylvania, USA.; 4 Division of Preventive Medicine, Department of Medicine, University of Alabama at Birmingham, Birmingham, Alabama, USA.; 5 Brigham and Women’s Hospital and Harvard Medical School, Boston, Massachusetts, USA.

**Keywords:** Immunology, Pulmonology, Complex traits, Genetic variation, NK cells

## Abstract

A role for hereditary influences in the susceptibility for chronic obstructive pulmonary disease (COPD) is widely recognized. Cytotoxic lymphocytes are implicated in COPD pathogenesis, and functions of these leukocytes are modulated by interactions between their killer cell Ig-like receptors (KIR) and human leukocyte antigen–Class I (HLA–Class I) molecules on target cells. We hypothesized HLA–Class I and KIR inheritance affect risks for COPD. HLA–Class I alleles and KIR genotypes were defined by candidate gene analyses in multiple cohorts of patients with COPD (total *n* = 392) and control smokers with normal spirometry (total *n* = 342). Compared with controls, patients with COPD had overrepresentations of HLA-C*07 and activating *KIR2DS1*, with underrepresentations of HLA-C*12. Particular HLA-KIR permutations were synergistic; e.g., the presence of HLA-C*07 + KIR2DS1 + HLA-C12^null^ versus HLAC*07^null^ + *KIR2DS1*^null^ + *HLA-C12* was associated with COPD, especially among HLA-C1 allotype homozygotes. Cytotoxicity of COPD lymphocytes was more enhanced by KIR stimulation than those of controls and was correlated with lung function. These data show HLA-C and KIR polymorphisms strongly influence COPD susceptibility and highlight the importance of lymphocyte-mediated cytotoxicity in COPD pathogenesis. Findings here also indicate that HLA-KIR typing could stratify at-risk patients and raise possibilities that HLA-KIR axis modulation may have therapeutic potential.

## Introduction

Chronic obstructive pulmonary disease (COPD) is a leading cause of death worldwide, and the prevalence, cumulative morbidity, and public health burden of this syndrome continue to increase (1). While tobacco smoking is the major predisposing factor for COPD in industrialized societies, genetic modifiers also appear important since only a minority of heavy smokers develop clinically significant lung disease, and familial clustering of cases is prominent (2, 3). Some genetic determinants of COPD have been identified by genome-wide association studies (GWAS) or other methods (3–5). However, the functional correlates of many disease-associated polymorphisms remain unknown, and variants reported to date account for only a small portion of COPD heritability (3, 5, 6). A better understanding of mechanisms that promote the development and/or progression of COPD could have significant, actionable importance.

Although many details of COPD pathogenesis remain enigmatic, the participation of cytotoxic lymphocytes (i.e., NK cells and some T cell subpopulations) in the causality and/or progression of this disease is widely acknowledged (6, 7). Human leukocyte antigens (HLAs) are the strongest known genetic determinants of immunologically mediated diseases and key regulators of cytotoxic lymphocytes (8). In particular, HLA–Class I molecules mediate antigen presentation to cytotoxic CD8^+^ T cells (9) and are cognate ligands for killer cell Ig-like receptors (KIR) on the surfaces of NK cells (10–12). NK cells can be either stimulated or inhibited by KIR crosslinking, depending on the structural characteristics of these receptors (13). Structure is also reflected in KIR nomenclature: the number of their extracellular domains (either 2 or 3) are designated as 2D or 3D, respectively, and the defining features of their cytoplasmic tails are denoted as either short (S) (which tend to be stimulatory) or long (L) (which are mostly inhibitory) (13). Hence, HLA engagements with various KIR can either stimulate or inhibit effector functions of NK cells and cytotoxic lymphocytes, depending on the relative expressions of particular KIR (10–12). Furthermore, specific HLA-KIR combinations modify the risks of developing several immunological syndromes, including diverse autoimmune diseases, malignancies, and infections (11, 12, 14).

We initially hypothesized that HLA–Class I polymorphisms may be important in COPD pathogenesis, similar to findings in other immune disorders (8, 9). We further presupposed disease-associated HLA biases would be most evident in focused candidate gene assays that compared extreme-of-phenotype cases — i.e., patients with COPD — with severe expiratory airflow versus control subjects with extensive smoking histories but normal spirometry.

Early findings of these assays, which showed HLA–Class I polymorphisms at the HLA-C locus are linked to COPD, prompted us to further examine the KIR that could interact with those alleles (10–14). In addition, since some actions of the HLA-KIR axis are known, it also seemed possible that these genetic findings might be amenable to additional corroboration by in vitro functional assays.

The results of these studies show that distinct HLA-C and KIR frequencies are biased in patients with COPD. In addition, the pathophysiologic effects of this phenomenon appear largely mediated via cytotoxic lymphocytes — and NK cells, especially.

## Results

### Discovery cohort subjects.

Smoking exposures and ages of the COPD subjects were slightly, but significantly, less than the smoke controls (SC) (participants who had at least 30 pack-years of cigarette smoking but normal spirometry) (Supplemental Table 1; supplemental material available online with this article; https://doi.org/10.1172/jci.insight.150187DS1). In total, 97% of the COPD subjects had severe or very severe lung disease (GOLD Stage 3 or 4) (1).

### Discovery cohort HLA–Class I allele prevalence.

An initial pilot trial comparing HLA–Class I alleles among the 170 COPD lung transplant subjects versus the first evaluable cohort of SC subjects (*n* = 81) indicated that intergroup differences were greatest at the HLA-C locus, especially at HLA-C*07 (Supplemental Table 2). An overrepresentation of HLA-B*07 in the COPD was less significant (Supplemental Table 2) and a likely feature of the interdependence of that allele with HLA-C*07, given that these 2 alleles are in strong linkage disequilibrium (LD) (i.e., an association of alleles at neighboring loci that exceeds purely random cosegregation) (15). HLA-C*07 is also more prevalent in the general population (16) and, hence, was presumed to have greater potential biological/clinical importance. Furthermore, there were no significant underrepresentations (a potentially protective polymorphism) of any HLA-A or HLA-B allele in the COPD, whereas there was a trend for decreased HLA-C*12 prevalence among the pilot trial SC subjects (Supplemental Table 2). Given these data, assays in the remaining subjects focused on definitions of HLA-C alleles.

The most significant findings in the analyses of the total discovery cohort (Supplemental Table 3) were an overrepresentation among the COPD of HLA-C*07 (OR = 2.32; 95% CI, 1.51–3.58; *P* = 0.0001) with an underrepresentation in these participants of HLA-C*12 (OR = 0.45; 95% CI, 0.25–0.95; *P* = 0.03).

### Validation cohort analyses.

SC in the validation cohort were younger than the comparison COPD subjects, whereas smoking exposures were not significantly different between these subpopulations (Supplemental Table 4). Validation COPD subjects had significantly less severe lung disease than the initial discovery cohort COPD transplant patients, as evidenced by percentages of predicted values for forced expiratory volume in 1 second (FEV_1_%p) of 36.3 ± 22.0 versus 25.2 ± 11.2, respectively (*P* < 0.0001) (Supplemental Tables 1 and 4).

The most striking intergroup differences of HLA-C allele frequencies within the validation cohort were, again, overrepresentations of HLA-C*07 (OR = 1.82; 95% CI, 1.21–2.75; *P* = 0.004) and underrepresentations of HLA-C*12 (OR = 0.50; 95% CI, 0.26–0.97; *P* < 0.04) among the COPD participants.

### HLA-C analyses of all subjects.

Table 1 details the demographic and clinical characteristics of the aggregate (all discovery and validation) subjects.

**Figure 1:**
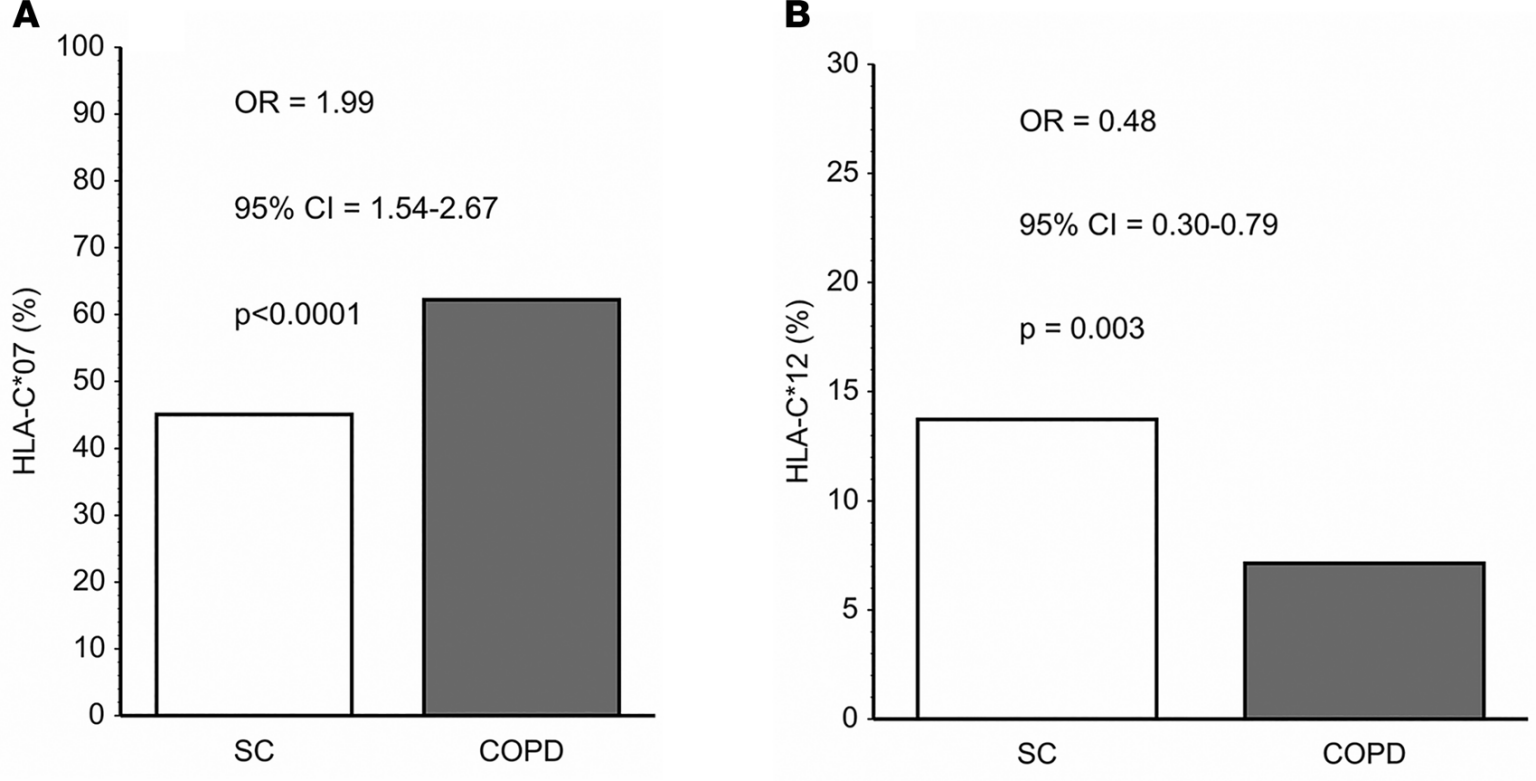
HLA-C*07 and HLA-C*12 are associated with COPD prevalence. (**A**) HLA-C*07 is overrepresented in the cumulative (discovery and validation) COPD cohort (*n* = 392) compared with the smoke controls (SC) (*n* = 342). Data are presented here (and elsewhere) as allele prevalence (the percentages of subjects with one or more copies of the allele). (**B**) HLA-C*12 was under-represented in the SC, compared with the COPD subjects. Comparisons were made by χ^2^ with determinations of OR and CI by logistic regression.

HLA-C allele analyses of this cumulative study population again showed that HLA-C*07 and HLA-C*12 had the strongest associations with COPD (Table 2 and Figure 1, A and B). COPD prevalence among HLA-C*07 + HLA-C*12 heterozygotes (46%) tended to be intermediate between subjects with only HLA-C*07 (62%) or only HLA-C*12 (35%), but the difference did not reach significance (*P* = 0.1). HLA-C*07 copy number did not alter COPD prevalence (61% versus 63%, for the 315 HLA-C*07 heterozygotes and 81 homozygotes, respectively; *P* = 0.7). Only 1 subject, a SC, was homozygous for HLA-C*12. Expiratory airflow obstruction was too strongly codependent with radiographic emphysema in the patients with COPD to allow meaningful assessments of HLA-emphysema associations in that subpopulation (FEV1%p versus emphysema score *P* < 0.0001). Emphysema was also present in 31% of SC, and neither HLA-C*07 nor HLA-C*12 was significantly associated with the presence or severity of this abnormality (data not shown).

### KIR genotypes.

A pilot analysis of KIR genotypes known to engage HLA-C alleles (11–14) indicated that the greatest intergroup difference among them was an overrepresentation of activating *KIR2DS1* in the COPD subjects (Supplemental Figure 1). *KIR2DS1* assays were performed in 349 COPD and 342 SC specimens with sufficient DNA. These assays confirmed that *KIR2DS1* was more prevalent in COPD than among SC (49.3% versus 29.8%, respectively; OR = 1.65; 95% CI, 1.21–2.26; *P* = 0.0017).

**Figure 2:**
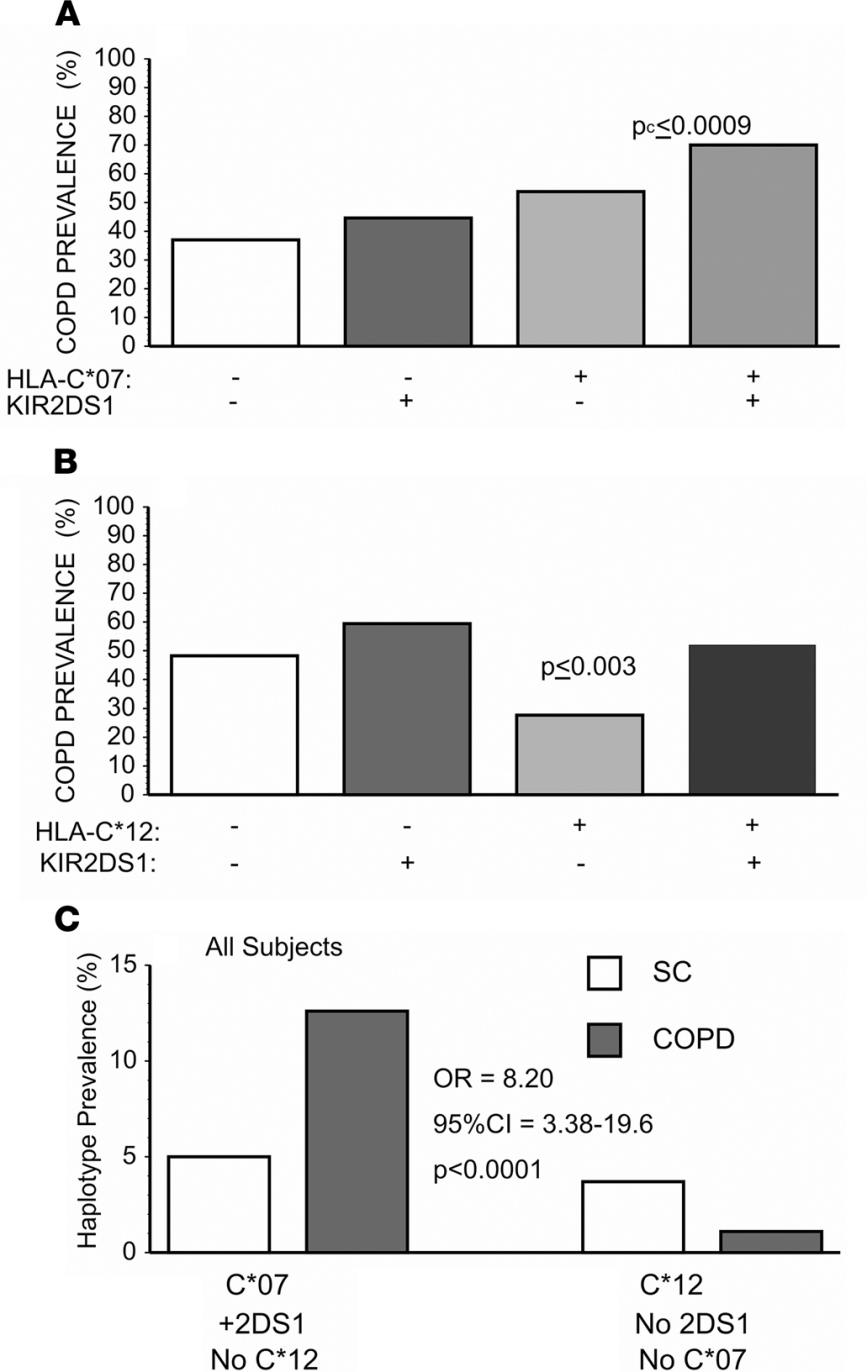
Interactive associations of HLA-C*07 and HLA-C*12 with *KIR2DS1* in COPD. (**A**) The prevalence of COPD among participants positive for both HLA-C*07 and *KIR2DS1* (*n* = 133) was significantly greater than in all the other subpopulations shown here. Analyses by χ^2^ with Bonferroni corrections for multiple comparisons (*P*_c_). (**B**) Conversely, the presence of HLA-C*12 in the absence of *KIR2DS1* (*n* = 46) appeared protective for COPD (*P*_c_ = comparisons of HLA-C*12 + *KIR2DS1*^null^ versus all other groups by χ^2^ with Bonferroni corrections). (**C**) The association of COPD with the highest risk haplotype (HLA-C*07 and *KIR2DS1* without HLA-C*12) was much greater than among subjects with the lowest risk haplotype (HLA-C*12 without either HLA-C*07 or *KIR2DS1*). Comparisons were made by χ^2^ with determinations of OR and CI by logistic regression. SC, smoke controls.

*KIR2DS1* also had interactive effects with both HLA-C*07 and HLA-C*12. The concomitant presence of *KIR2DS1* among subjects with HLA-C*07 multiplied risks for COPD (Figure 2A). Conversely, the presence of *KIR2DS1* seemed to largely negate COPD “protection” associated with HLA-C*12 (Figure 2B). Given the potential complexities of these interactions, we compared COPD prevalence among the subpopulation with the highest disease risk (HLA-C*07 + *KIR2DS1* + HLA-C*12^null^) versus those with the least risk (HLA-C*12 + HLA-C*07^null^ + *KIR2DS1*^null^) and again found strong epistatic (multiplicative) effects among these genetic elements (Figure 2C).

**Figure 3:**
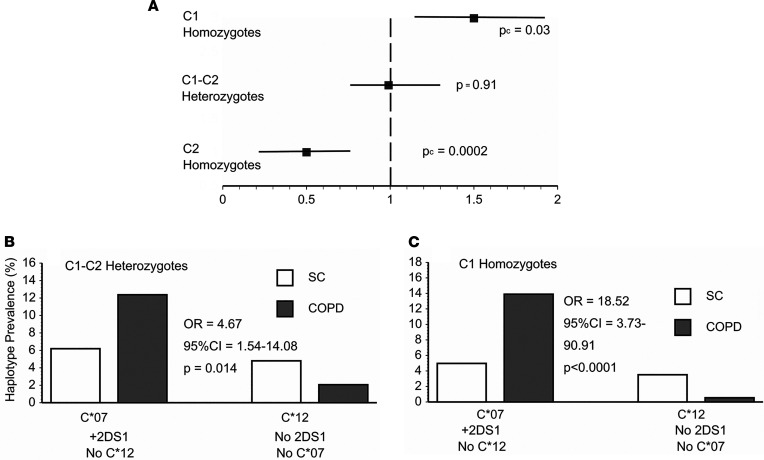
COPD associations with HLA-C allotypes. (**A**) COPD prevalence was greatest in subjects homozygous for HLA C1 allotypes (*n* = 307) (Table 2) than among C1-C2 allotype heterozygotes (*n* = 315), and it was least among those homozygous for C2 allotype alleles (*n* = 116). Bars depict 95% CI, and solid squares denote OR for the presence of COPD. Analyses by χ^2^, with Bonferroni corrections for multiple comparisons. OR and CI determined by logistic regression. (**B**) COPD associations with highest risk haplotype (HLA-C*07 *+ KIR2DS1* + HLA-C*12^null^) (*n* = 123) versus lowest risk haplotype (HLA-C*12 in the absence of either HLA-C*07 or *KIR2DS1*) (*n* = 34) were less striking in C1-C2 allotype heterozygotes (*n* = 315), compared with similar analyses of the entire subject population (Figure 2C). (**C**) COPD associations in highest risk versus lowest risk haplotypes were especially strong among C1 homozygotes.

Functional effects of HLA-KIR interactions can vary highly depending on which of the dimorphic HLA-C allotypes, determined by polymorphisms at positions 77 and 80 of the C-terminal heavy chain, are involved in that engagement (10–14). HLA-C1 allotypes (serine 77, asparagine 80) include HLA-C*07 and HLA-C*12, whereas HLA-C2 allotypes have asparagine in position 77 and lysine at 80 (Table 2). Because these HLA-C allotypes may exert profoundly different actions, and/or have distinct disease distributions (10–14), we examined COPD associations with the 2 HLA-C allotypes. COPD was much more prevalent in C1 homozygotes (59%) than among HLA-C2 homozygotes (38.8%) (Figure 3A). Even more striking, the interactive effects of HLA alleles C*07 and C*12 with *KIR2DS1* were also highly modified among the different HLA-C allotypes, with the strongest epistasis evident in C1 allotype homozygotes (Figure 3, B and C).

### Peripheral blood mononuclear cell (PBMNC) phenotypes.

**Figure 4:**
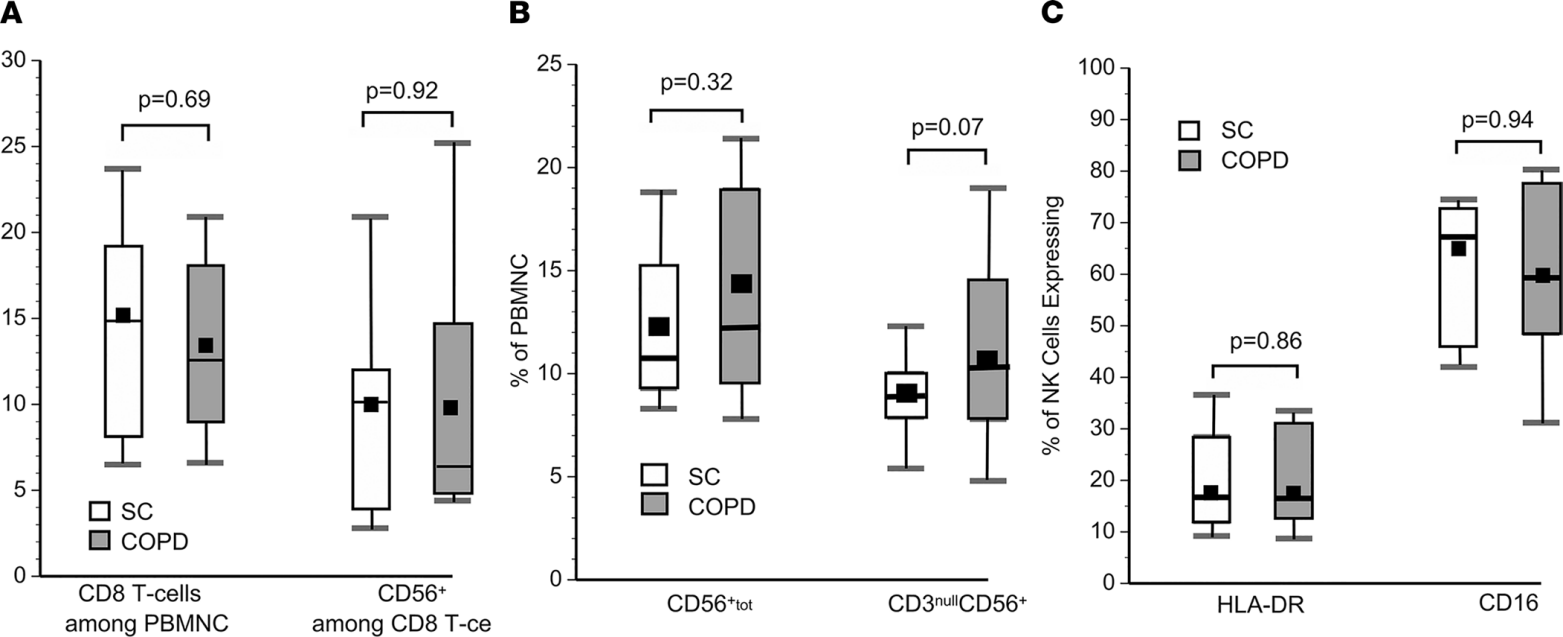
Cytotoxic lymphocyte phenotypes. (**A**) COPD and smoke control (SC) subjects (*n* = 26 in each group) did not differ in the proportions of CD8^+^ T cells among their peripheral blood mononuclear cells (PBMNCs) or CD8^+^CD56^+^ (putative NKT cells). The lowest, second lowest, middle, second highest, and highest lines represent 10th, 25th, 50th, 75th, and 90th percentiles, respectively. The mean is denoted with a closed square. (**B**) There was a nonsignificant trend for larger proportions of CD3^null^CD56^+^ (NK cells) among the PBMNC of the COPD subjects. (**C**) The extents of activation (HLA-DR^+^) and Fc receptor CD16 expression on NK cells (CD3^null^CD56^+^) were similar in both subject populations. Comparisons were made by Mann-Whitney *U* tests.

The apparent associations of COPD with the HLA-KIR axis, and the presumed importance of these genetic interactions in cytotoxic lymphocyte functions, prompted further studies to investigate these lymphocytes in the subject subpopulations. We first found the proportions of cytotoxic CD8^+^ T cells were similar in both subject groups (Figure 4A). Similarly, the proportion of PBMNC that expressed NK cell marker CD56 did not significantly differ between subpopulations (Figure 4B). The proportions of NK cells (CD3^null^CD56^+^) that expressed activation marker HLA-DR and Fc receptor CD16 were also nearly identical in these groups (Figure 4C).

### Cytotoxic activity.

**Figure 5:**
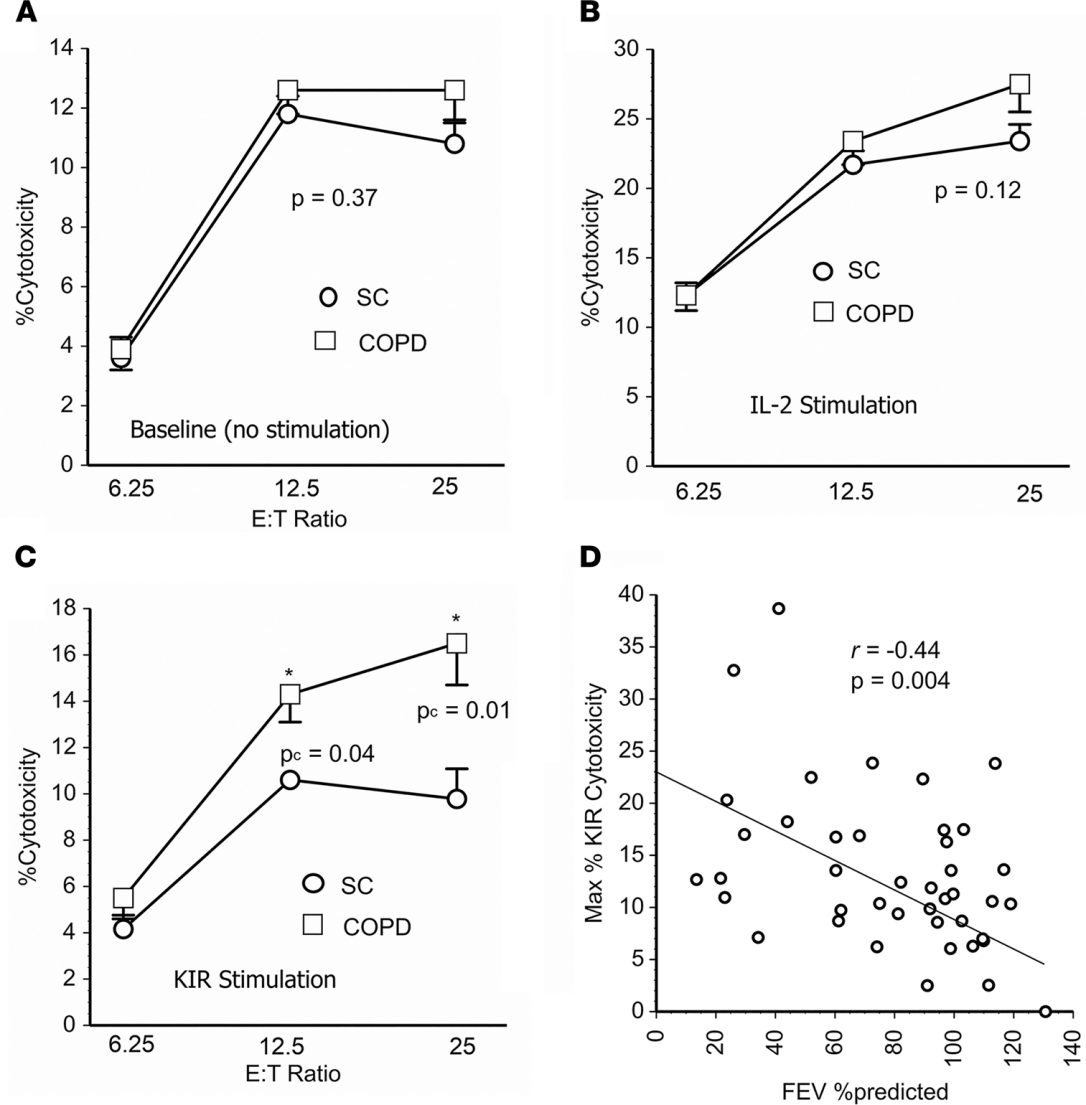
Activation of cytotoxic lymphocytes. (**A**) There were no differences of peripheral blood mononuclear cell (PBMNC) cytotoxic activity among smoke control (SC) and COPD specimens (*n* = 21 each) at baseline (without ex vivo stimulation) or after incubation with IL-2 (**B**) at varying ratios of effectors (PBMNC) to K562 targets (E:T ratios). Data are depicted as means ± SEM. Comparisons by Mann-Whitney *U* tests. (**C**) In contrast, prior incubation of PBMNC with a cocktail of anti-KIR antibodies resulted in greater cytotoxicity among COPD specimens. Comparisons made by Mann-Whitney *U* tests with Bonferroni corrections. (**D**) The maximal KIR-induced cytotoxicity (E:T = 25) in these cumulative specimens was inversely correlated (by Spearman’s correlation coefficient) with forced expiratory volume in 1 second (FEV_1_) as a percent of predicted normal values.

PBMNC of COPD and SC had similar cytotoxic activities at baseline and after incubation with IL-2, a nonspecific activator of NK cells and other lymphocytes (Figure 5, A and B). However, stimulation with anti-KIR antibodies, to recapitulate in vivo engagements of lymphocyte KIR with HLA ligands, increased cytolytic activity of PBMNC from the patients with COPD to a greater degree than that of the comparable SC preparations (Figure 5C).

The magnitude of cytolysis induced by in vitro KIR cross-linking was also inversely correlated with the pulmonary function of the individuals providing these specimens (Figure 5D). In contrast, PBMNC cytotoxicity at baseline and after nonspecific activation with IL-2 was not significantly associated with pulmonary function (*r* = 0.07 and *P* = 0.65, and *r* = 0.12 and *P* = 0.41, respectively).

**Figure 6:**
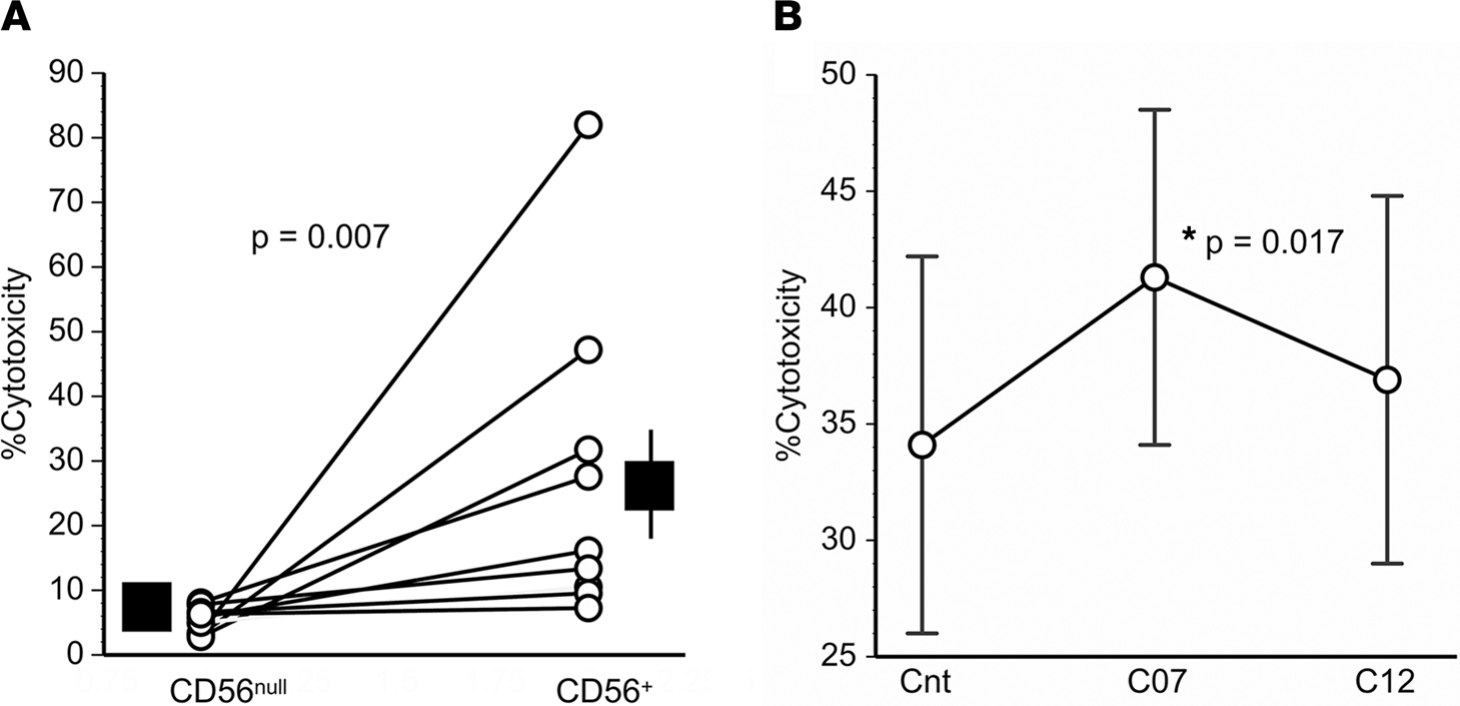
Differential lymphocyte cytotoxicity. (**A**) Cytolytic activity of peripheral blood mononuclear cells (PBMNC) depleted of NK cells (CD56^null^) was less than corresponding CD56^+^ cells (NK cells) isolated from these PBMNC in every paired specimen (*n* = 9). Effector (cytotoxic lymphocyte)/target (K562 cells) ratios ranging from 0.5 to 2.0 were tested (E:T ratios were identical between concomitant paired CD56^null^ and CD56^+^ coculture assays, connected by lines). Solid squares denote means, and vertical lines are SEM. Comparisons by Mann-Whitney *U* tests. (**B**) PBMNC cytolysis of K562 cells that expressed HLA-C*07 (C07) after transfection (*n* = 12) was much greater than K562 cells after mock transfections (Cnt) or among K562 cells that were transfected to express HLA-C*12 (C12) (*n* = 12 in each group). E:T = 20:1. Data are shown as mean ± SEM. Analyses by Friedman Statistic, with Student-Newman-Keuls for multiple comparisons.

To determine the subpopulation most responsible for cytotoxicity, analogous experiments were performed using either CD56^+^ (NK cells) or CD56^null^ subpopulations isolated from PBMNC, with their respective purities confirmed by flow cytometry (Supplemental Figure 2). Intergroup comparisons showed that the NK cells had much greater cytotoxic activity (Figure 6A).

Because the COPD associations of HLA-C*07 and HLA-C*12 were highly varied (Figures 1 and 2), even though both C1 allotypes nominally have similar KIR avidities (10–14), we hypothesized that engagements of these alleles may, in fact, evoke different NK cell responses. Accordingly, we generated K562 target cells that expressed either one or the other of these HLA alleles and used these as targets in cytotoxic assays. We found K562 cells transfected with HLA-C*07 were more susceptible to NK cell cytolysis than either sham-transfected target cells or those that expressed HLA-C*12 (Figure 6B).

## Discussion

The present studies show that certain immunoregulatory gene polymorphisms are highly associated with COPD susceptibility. In particular, HLA-C*07 was overrepresented among patients with COPD compared with smokers with normal pulmonary function (Figure 1A), whereas HLA-C*12 was conversely underrepresented among the patients with COPD (Figure 1B). Subsequent investigations revealed NK cell function–stimulating *KIR2DS1* is also overrepresented among COPD subjects (Figure 2B), and moreover, effects conferred by this gene are interactive with both HLA-C*07 and HLA-C*12 alleles (Figure 2, A–C). Stratifications to compare subjects with the highest risk haplotype (HLA-C*07 + *KIR2D1* + HLA-C*12^null^) versus those with the lowest risk haplotype (HLA-C*12 + *KIR2DS1*^null^ + HLA-C*07^null^) showed strong epistatic associations, especially among those who were homozygous for HLA-C1 allotypes (Figure 3C). Correlative functional assays showed that KIR-mediated cytolysis is greatest in patients with COPD (Figure 5C), significantly related to pulmonary physiology (Figure 5D), and enhanced among target cells that display HLA-C*07 compared with those expressing HLA-C*12 (Figure 6B). The present data support the concept that COPD pathogenesis results from a complex interplay between environmental agents (e.g., tobacco smoke) and multiple genetic determinants (3, 6). To the best of our knowledge, the series of findings presented here are unprecedented.

While the importance of immunological processes in COPD pathogenesis has long been appreciated (17), only a few studies have attempted to characterize HLA alleles among afflicted patients by use of specific, candidate gene assay methods (18–20). An early investigation used imprecise serologic typing to analyze a limited repertoire of HLA-A and -B alleles (many more alleles at each of these loci have since been discovered) and found HLA-B*07 was a susceptibility factor for COPD (18). That report may be concordant with the present data that also indicate a potential link between HLA-B*07 and COPD (Supplemental Table 2), although we believe our finding is likely due to the very strong LD between this allele and HLA-C*07 (15). However, a later investigation, limited to an examination of HLA-B*07 among patients with relatively mild COPD, did not substantiate the initial report (19). More recently, analyses of HLA–Class II DR and DQ allele frequencies in a single cohort study with 320 patients found overrepresentations of HLA-DRB1*14 in their patients with COPD (20). The present study differs significantly from these earlier reports in several aspects, such as the use here of larger numbers of highly characterized subjects in extreme-of-phenotype comparisons, as well as prospective study of distinct replication cohorts. Moreover, we are unaware of any previous candidate gene analyses of HLA-C (or KIR) in COPD or attempts to link those genetic elements with immunological functions.

HLA and KIR typing by techniques that target these candidate genes, such as PCR amplifications using sequence-specific primers (PCR-SSP), are definitive and may arguably be a method of choice for focused, finite pilot investigations (such as this study), but they are time-consuming, tedious, expensive, and utilize large amounts of precious specimen DNA. In contrast, high-throughput GWAS are facile and economical, especially in studies of large populations, and they concurrently identify many diverse disease-associated polymorphisms. Nonetheless, the study of HLA and KIR regions by GWAS can be problematic. The chromosomal regions for HLA (6p21) and KIR (19q13) are both characterized by unusually high gene densities, extreme polymorphism, and strong, nonrandom LD that complicate and confound GWAS imputations (21). Furthermore, discovery of synergistic (epistatic) interactions between disparate loci (e.g., HLA and KIR genes) may not be readily discerned by routine GWAS analyses. The COPD SNP variants dentified by GWAS so far are also often associated with low-level risks that account for little of the heritance of this complex syndrome and do not address phenotypic heterogeneity and disparate subject populations. In addition, the functional correlates of disease-associated variant SNPs, many of which are located in noncoding regions, may not be evident or inform understandings of underlying pathological mechanisms. Despite inherent limitations, however, GWAS have found COPD associations with SNP in the region that includes HLA-C (22, 23), and another disease susceptibility locus has been putatively identified in or proximate to the region encoding KIR (24, 25).

The present findings are also concordant with studies that implicate NK cells in COPD pathogenesis (7, 26–28). Although most previous reports find minimal differences of NK cell counts in peripheral blood of patients with COPD compared with controls (Figure 4) (29, 30), subtle phenotypic alterations that associate with disease manifestations have also been described (26). Functional NK cell alterations, including increased activation and cytotoxic activity, as well as correlations between these altered functions and disease severity, have been found among patients with COPD (29) and were also seen here (Figure 5, C and D).

Although the pathological importance of the HLA-KIR axis is recognized in many other syndromes (10–13, 31, 32), parsing out the nuances of this immunoregulatory mechanism is rendered difficult by many complexities and peculiarities. The net effect of the interactions mediated by the polymorphic KIR repertoire of any given NK cell depends on an integration of signals transduced from both function-inhibiting and function-stimulating receptors (10, 32). Further confounding is introduced by the varied cell surface expression of the KIR per se, being unpredictably present in varying densities and proportions of cytotoxic effectors (33); the extent of HLA-C surface expression on target cells is also often highly variable. The lack of monoclonal antibodies with unique specificities for individual KIR hinders direct quantitation of these receptor expressions and assessments of their specific functions (34). Additional intricacies of this immunomodulatory process can arise from “education” of NK cells during their maturation, wherein engagements of their KIR with autologous, cognate HLA ligands lead to NK cell hyporesponsiveness (analogous to induction of peripheral tolerance in T cells). *KIR2DS1* is relatively unique among function-stimulating KIR in that it may variously be involved in both “education” and activation (35). *KIR2DS1* binding affinity is nominally limited to HLA-C2 allotypes (10), in whom earlier “education” results in NK cell hyporeactivity. Conversely, however, NK cell activity may be enhanced by the presence of *KIR2DS1* among individuals who are C1/C2 heterozygotes and even more so among C1 allotype homozygotes (Figure 3) (13, 36). The findings in these patients with COPD are very analogous to reports that have similarly shown the HLA-KIR combinations of importance seen here (Figure 1, Figure 2, and Figure 3) are also associated with increased risks for the presence of other immunological syndromes (10–13, 31).

This was an initial survey using a definitive, if cumbersome, methodology to test our hypothesis that HLA–Class I alleles may be associated with COPD susceptibility. This study was designed to identify biases (if present) among the most prevalent HLA–Class I (and KIR) that have especially strong associations with COPD, by comparing populations with extreme phenotypic differences (Table 1). The findings of this pilot trial justify further study with more detailed and thorough genetic analytical methodologies, such as next-generation sequencing (NGS) (37). In-depth studies of the COPD genetic “hotspots” localized here will further characterize the involvement of HLA and KIR in COPD by more precise identifications of the particular allelic variants with disease associations (there are 17 alleles of 2DS1 alone; refs. 13, 38). NGS studies could also possibly detect other genes within or close to the HLA and KIR loci that also contribute — if more weakly — to lung disease development and/or progression. In addition, the use of detailed high-throughput genetic analyses will expedite the incremental study of other demographic groups. The exclusive study of non-Hispanic White individuals in the present study limits the generalizability of these results since both HLA and KIR regions are associated with large differences between lineages (16, 39).

The development of facile tests that accurately identify patients at heightened risk for COPD could have several important clinical applications, including enriched selections of subjects with the greatest disease predilections for expensive clinical trials and personalized counseling and/or increased surveillances of especially at-risk individuals and their family members. Perhaps more importantly, better understanding of the pathological mechanisms of COPD might illuminate approaches for innovative therapies and perhaps eventually guide the selection of biological response modifiers that specifically (and more effectively) target the causal processes of this recalcitrant lung disease.

## Methods

Supplemental Methods are available online with this article.

### Participants.

The initial COPD discovery cohort consisted of patients who underwent lung transplantation evaluations at the University of Pittsburgh Medical Center (UPMC) and had fixed expiratory airflow obstruction due to tobacco smoking. Discovery cohort SC were participants in the UPMC Specialized Centers of Clinically Oriented Research registry (SCCOR) who had at least 30 pack-years of cigarette smoking, but normal spirometry.

The validation cohort included Temple University Medical Center patients with severe airflow obstruction (i.e., GOLD Stage 3 or 4) (1), as well as consecutive SCCOR COPD participants. Validation SC subjects were recruited from the Pittsburgh Lung Screening Study registry at the Pittsburgh Cancer Institute. All validation participants fulfilled the inclusion and exclusion criteria of their respective discovery subpopulations. SCCOR participants (both COPD and SC) had high-resolution chest CT evaluations to evaluate emphysema (40).

Analyses were restricted to non-Hispanic White individuals because fewer than 5% of the initial discovery COPD lung transplantation cohort were members of underrepresented minority groups, and HLA allele frequencies vary among racial and ethnic subpopulations (13, 41).

### HLA typing.

HLA were defined by analyses of leukocyte DNA using sequence-specific oligonucleotide probe assays, or PCR-SSP. These and other assays in this study were conducted by laboratory personnel who were blinded to subject identities and characteristics.

### KIR allele typing.

KIR genes were detected by PCR-SSP using methods previously published (41).

### Cytotoxic cell assays.

PBMNC were characterized by flow cytometry, using methods detailed elsewhere (42). PBMNC (effector cells) were used in cytotoxicity assays after 2 days treatment in: (a) media control (baseline); (b) media supplemented with IL-2 at 6000 IU/mL; or (c) wells precoated with mixtures of an anti-KIR monoclonal antibody cocktail (MAB1848 and MAB2238, R&D Systems, Bio-Techne; and HP-MA4, BioLegend). Cytolysis of K562 cells labeled with ^3^H-thymidine or calcein was detected by β-counter (40) or flow cytometry (43), respectively. In some cases, PBMNC were segregated prior to assays into CD56^+^ and CD56^null^ subpopulations using immunomagnetic beads and/or FACS.

### Target cell HLA-C transfections.

HLA-C*07 and HLA-C*12 were expressed in HLA^null^ K562 target cells after cloning with a lentivirus construct system. PCR, sequencing, and flow cytometry confirmed successful HLA-C transfection and expression in the target cells (Supplemental Figure 3).

### Statistics.

HLA allele and KIR gene prevalences are described as the percentages of subjects with one or more copies of these genetic element(s). Intergroup comparisons of these genetic elements were made by χ^2^, with logistic regression used to generate OR and CI. Ordered and continuous variables were compared by Mann-Whitney *U* rank-sum tests. Friedman tests were used to compare effects of multiple treatments in the same specimens. Associations between 2 continuous variables were examined by Pearson product-moment correlations. *P* values corrected for multiple comparisons by Bonferroni are denoted as *P*_c_. *P* (or *P*_c_ if applicable) < 0.05 was considered significant. Unless specified otherwise, data are depicted as means ± SEM.

### Study approval.

Written informed consent was received from all participants prior to inclusion in the study. Research protocols were approved by the IRBs at the University of Pittsburgh, Temple University, and University of Alabama at Birmingham.

## Author contributions

All authors contributed to the writing and/or editing of this manuscript. Conception and design were contributed by SRD, YZ, and FCS; acquisition of data for the work was contributed by TM, TKTN, KY, YZ, JX, JMP, and GJC; analysis and interpretation were contributed by TM, YZ, YK, and SRD; and drafting the manuscript for important intellectual content was contributed by TM, AG, YK, MHC, and SRD.

## Supplementary Material

Supplemental data

## Figures and Tables

**Table 1:**
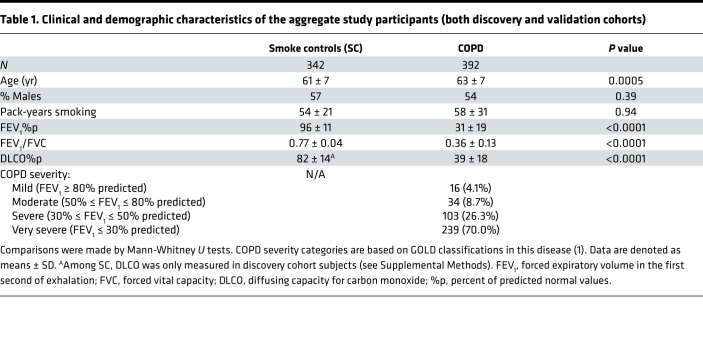
Clinical and demographic characteristics of the aggregate study participants (both discovery and validation cohorts)

**Table 2:**
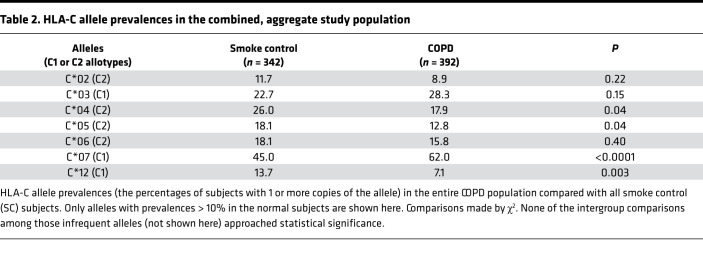
HLA-C allele prevalences in the combined, aggregate study population

## Version 1 08/31/2021

In-Press Preview

## Version 2 10/08/2021

Electronic publication
