# Supplementary material for: HLA-C and KIR permutations influence chronic obstructive pulmonary disease risk
Source: JCI Insight. 2021 Oct 8;6(19):e150187. doi: 10.1172/jci.insight.150187 (PMC8525585; doi:10.1172/jci.insight.150187)
Supplement: Supplemental data [file jciinsight-6-150187-s115.pdf]

ONLINE SUPPLEMENTS:

HLA-C AND KIR PERMUTATIONS INFLUENCE CHRONIC OBSTRUCTIVE PULMONARY DISEASE RISKS

Takudzwa Mkorombindo, M.D., Thi K. Tran-Nguyen, Ph.D., Kaiyu Yuan, M.D.,

Yingze Zhang, Ph.D., Jianmin Xue, M.D., Gerald J. Criner, M.D., Joseph M. Pilewski, M.D.,

Amit Gaggar, M.D., Michael H. Cho, M.D., Frank C. Sciurba, M.D., Steven R. Duncan, M.D.

ONLINE SUPPLEMENT 1

Methodological Details

HLA Typing: HLA characterizations of the initial lung transplantation recipient discovery cohort were performed by the clinical laboratory at the University of Pittsburgh Medical Center (UPMC), in DNA isolated from leukocytes, using sequence-specific oligonucleotide probe assays (Dynal RELI™ SSO, Invitrogen).

Polymerase chain reaction (PCR) sequence-specific primer (SSP) amplifications (AllSet+™ Gold, Life Technologies) were used to define HLA Class I alleles in the other subjects. Because a pilot assay showed the greatest intergroup differences were among HLA-C alleles (see below), typing in subsequent subjects was limited to this locus.

KIR Allele Typing: KIR genes were detected by PCR-SSP of leukocyte DNA using primers and methods previously detailed (1).

Cytotoxic Cell Characterizations and Functional Assays: Peripheral blood mononuclear cells (PBMNC) were isolated from venous phlebotomy specimens by Ficoll density gradient centrifugation. PBMNC from consecutive SCCOR subjects (both COPD and SC) were phenotyped by flow cytometry, using reagents, equipment, and methods detailed previously (2).

The initial series of cytotoxicity assays used PBMNC (effector cells) from COPD or SC subjects that had been cultured in complete media (RPMI1640, 10% human AB sera, pen/strep, L-glutamine, HEPES) at $1.5x10^6$ cells/ml in one of three conditions: 1) otherwise untreated media (baseline); 2) media supplemented with IL-2 at 6000 IU/ml (ProSpec); or 3) in wells pre-coated with mixtures (2 µg/ml each) of anti-KIR monoclonal antibodies (MAB1848 and MAB2238, R & D Systems and HP-MA4, BioLegend). After two days of these treatments, the effector cells were subsequently co-cultured for four hours with K562 target cells that had been radiolabeled during the

previous 18 hours with ^{3}H-thymidine, in varying effector:target (E:T) cell ratios (0:1, 6.25:1, 12.5:1, 25:1). Counts per minute (cpm) were determined in a β-counter as a measure of intact residual (unfragmented) target cell DNA (2). Cytotoxicity% was calculated as $[(S_{cpm} – E_{cpm})/S_{cpm}] \times 100$ where S= control spontaneous release wells (target cells only) and E=experimental wells with admixed treated effector and target cells (2).

In subsequent experiments to determine the effector cell(s) most responsible for cytotoxicity, PBMNC were segregated into CD56$^+$ and CD56^{null} subpopulations prior to their use in cytotoxicity assays. The CD56$^+$ cells were isolated by negative selection using a microbead NK cell isolation kit (Miltenyi Biotec), followed by an additional T-cell depletion with anti-CD3 microbeads (Miltenyi Biotec). CD56^{null} cells were obtained by fluorescence-activated-cell-sorting (FACS) depletion of leukocytes that stained with CD56-PE (BD Biosciences) and FACS (BD FACSAria, BD Bioscience) in other aliquots of PBMNC. The effector cell subpopulations (either CD56$^+$ or CD56^{null}) were individually incubated in IL-2 (6000 IU/ml) for two days, and then co-cultured for 4 hours with K562 target cells that had been previously labeled with calcein AM (0.1 µg/ml) (R&D Biosciences) for 2 minutes (3). Pilot study indicated the extent of cytotoxicity with the CD56$^+$ effectors was much greater than in the initial assays that used unsegregated PBMNC. Accordingly, E:T ratios for these particular assays were 0.5:1, 1:1, and 2:1. The labeled K562 target cells (calcein$^+$) were characterized after four hours of co-culture by their uptake of 7-amino-actinomycin D (7-AAD), ascertained by flow cytometry, and are reported as percentages of the total live (7-AAD^{null}) vs. dead/dying (7-AAD$^+$) K562 cells.

Target Cells Transfections with HLA-C*07 or HLA–C*12: Total RNA was extracted using Trizol reagent (ThermoFisher, Waltham, MA) from PBMNC cells of COPD patients that were known to have either HLA-C*07 or HLA-C*12 by prior PCR-SSP typing. cDNA in these specimens was synthesized by oligo-DT priming and a SuperScript IV First-Stand Synthesis System (ThermoFisher). HLA-C-specific sequences were amplified by PCR with KOD Hot Start DNA

polymerase (Millipore Sigma) and with Forward primer: 5'–AGCTGAACCGGTATGCGGGTCATG GCG CCCCGAGCCCTC-3'; and Reverse Primer: 5'–AGCTGAGAATTCTCAGGCTTTACAAGTGA GAGAGACTC-3'. The forward primer was designed with an *AgeI* restriction endonuclease site and the reverse primer incorporated an *EcoRI* site.

A lentiviral construct pLJM1-EGFP (Addgene) was digested with AgeI and EcoRI to cut out the enhanced green fluorescent protein gene, and the products of the HLA-C*07 and HLA-C*12 PCRs were similarly treated. DNA fragments resulting from these digests were electrophoresed in 1% agarose, and bands corresponding to the appropriate length products were cut out and purified using Qiaquick PCR purification Kit (Qiagen). The HLA-C*07 and HLA-C*12 DNA were individually treated with ligase (New England Biolabs), along with lentivirus construct fragments, and the resultant products used to transform One Shot STBL3 Chemically Competent Cells (ThermoFisher). DNA was extracted from single clones and sequenced to confirm insertion of pLJMI and either full-length HLA-C*0702 or HLA-C*1203. Lentiviruses were produced by transient transfection of HEK293T cells with these constructs, along with pCMV-VSVG and pCMV-dR8.2 dvpr plasmids (Addgene), using Lipofectamine 2000 (ThermoFisher). K562 cells (which do not express HLA) were infected with the lentivirus-like particles and selected using purimycin (2ug/ml) (Sigma). The presence of HLA-C*07 or HLA-C*12 mRNA in K562 clones was confirmed by SSP-PCR, and surface expression demonstrated by flow cytometry after staining with anti-HLA-C-PE mAb (BD Biosciences). K562 cells that expressed HLA-C*07 or HLA-C*12, along with mock-transfected cells (controls), were used as targets in cytotoxicity assays, as detailed above.

Antibodies Utilized: Antibodies used for flow cytometry: CD3 (BD Biosciences #347344 & 555342), CD 8 (BD Biosciences #555369 & 340046), CD16 (BD Biosciences #555404), CD 56 (BD Bioscience #557747), anti-HLA-DR (BD Biosciences #555811), anti-HLA-C (BD Biosciences #566372), anti-human CD158 (Biolegend # 339506, R & D Systems #MAB1848 & #MAB2238), .

Data were acquired on a BD LSR-II Cytometer (BD Biosciences) and analyzed by FlowJo software (FlowJo).

Statistical Methods: HLA allele and KIR gene prevalences were calculated as the percentages of subjects with one or more copies of these particular genetic element(s). Comparisons of ordered and continuous variables were made by Kruskal-Wallis tests. Dichotomous variables were compared by chi-square, and logistic regression was used to generate odds ratios (OR) and confidence intervals (CI). Relationships between continuous variables were established by linear regression. Friedman tests were used to compare effects of three treatments in the same specimens. P values <0.05 were considered significant. Unless specified otherwise, data are denoted as means $\pm$ SD. Data analyses were conducted in STATA version 13.0 (StataCorp) or StatView version 5.0.1 (SAS Institute Inc., Cary) by TM, YZ, and SRD.

References:

1. Martin MP and Carrington M. KIR locus polymorphisms: genotyping and disease association analysis. *Methods Mol Biol* 2008; 415: 49-64.
2. Feghali-Bostwick CA, et al. Autoantibodies in patients with chronic obstructive pulmonary disease. *Am J Respir Crit Care Med* 2008; 177: 156-163.
3. Neri S, et al. Calcein-acetyoxymethyl cytotoxicity assay: standardization of a method allowing additional analyses on recovered effector cells and supernatants. *Clin Diagn Lab Immunol* 2001; 8: 1131-1135.

Tables

Online Supplement Table 1.

Clinical and Demographic Characteristics of the Discovery Cohort

	Smoke Controls	COPD	p value
N	180	170	
Age (yr)	64.4 $\pm$ 5.8	62.1 $\pm$ 6.5	**0.047**
% Males	53	50	0.53
Pack-years smoking	55.4 $\pm$ 25.4	50.2 $\pm$ 23.6	**0.034**
FEV_1%p	98.0 $\pm$ 10.7	25.2 $\pm$ 11.2	**<0.0001**
FEV_1/FVC	0.77 $\pm$ 0.04	0.33 $\pm$ 0.10	**<0.0001**
DLCO%p	81.7 $\pm$ 13.6	33.0 $\pm$ 14.4	**<0.0001**
GOLD Stages[1]:	NA		
One		1 (0.6%)	
Two		4 (2.4%)	
Three		40 (23.5%)	
Four		125 (73.5%)	

FEV_1 = forced expiratory volume in the first second of exhalation; FVC = forced vital capacity; DLCO = diffusing capacity for carbon monoxide; %p = percent of predicted normal values. NA = not applicable. GOLD stages correspond to COPD severity, with increments denoting progressively worse disease (1). Data are denoted as means $\pm$ SD.

Reference:

1. Singh D, et al. Global strategy for the diagnosis, management, and prevention of chronic obstructive lung disease: the GOLD science committee report 2019. *European Respiratory Journal* 2019; 53: 1900164

Online Supplement Table 2

HLA Class I Allele Prevalences in the Initial Pilot Trial

**Alleles**	**Smoke Control (n = 81)**	**COPD (n = 170)**	**p**
A*01	33.3	34.1	n.s.
A*02	49.4	44.7	n.s.
A*03	24.7	28.8	n.s.
A*11	11.1	9.4	n.s.
A*24	14.8	14.7	n.s.
A*32	11.1	8.2	n.s.
B*07	13.6	31.8	0.002
B*08	22.2	27.6	n.s.
B*27	12.3	8.2	n.s.
B*35	24.7	15.9	n.s.
B*44	23.5	24.1	n.s.
C*03	25.9	24.1	n.s.
C*04	27.2	19.4	n.s.
C*05	19.7	12.9	n.s.
C*06	14.8	18.8	n.s.
C*07	38.3	67.1	<0.0001
C*12	13.6	6.5	0.06

HLA Class I allele prevalences (the percentages of subjects with one or more copies of the allele) in the initial COPD transplant recipient population were compared to results in the initial Smoke Control

(SC) subjects. These findings prompted further study of specimens collected from subsequent SC for comparisons, while focusing on analyses of HLA-C polymorphisms. Only alleles with prevalences >10% in the normal subjects are shown here.

Online Supplement Table 3

HLA-C Allele Prevalences in the Entire Discovery Cohort

**Alleles**	**Smoke Control (n = 180)**	**COPD (n = 170)**	**p**
C*03	25.0	24.1	n.s.
C*04	25.0	19.4	n.s.
C*05	18.3	12.9	n.s.
C*06	16.7	18.8	n.s.
C*07	46.7	67.1	0.0001
C*12	13.3	6.5	0.03

HLA Class I allele prevalences (the percentages of subjects with one or more copies of the allele) in the discovery COPD population compared to all discovery Smoke Control (SC) subjects. Only alleles with prevalences >10% in the normal subjects are shown here. None of the intergroup comparisons among those infrequent alleles (not shown here) approached statistical significance.

Online Supplement Table 4

Characteristics of the Validation Cohort

	Smoke Controls	COPD	p value
N	162	222	
Age (yr)	57.8 $\pm$ 6.3	62.9 $\pm$ 7.0	**<0.0001**
% Males	60	56	0.41
Pack-years smoking	53.2 $\pm$ 14.7	63.7 $\pm$ 34.9	0.09
FEV_1%p	94.7 $\pm$ 10.8	36.3 $\pm$ 22.0	**<0.0001**
FEV_1/FVC	0.76 $\pm$ 0.04	0.37 $\pm$ 0.14	**<0.0001**
DLCO%p	ND	44.7 $\pm$ 19.8	
GOLD Stages:	NA		
One		15 (6.8%)	
Two		30 (13.5%)	
Three		68 (28.4%)	
Four		114 (51.4%)	

FEV_1 = forced expiratory volume in the first second of exhalation; FVC = forced vital capacity; DLCO = diffusing capacity for carbon monoxide; %p = percent of predicted normal values. ND = not done; NA = not applicable. GOLD stages correspond to the extent of COPD severity, with increments denoting progressively worse disease (1). Data are denoted as means $\pm$ SD.

Reference: 1. Singh D, et al. Global strategy for the diagnosis, management, and prevention of chronic obstructive lung disease: the GOLD science committee report 2019. *Eur Respir J.* 2019; 53: 1900164

Figures

Online Supplement Figure 1

Pilot Study Analysis of KIR Gene Prevalences

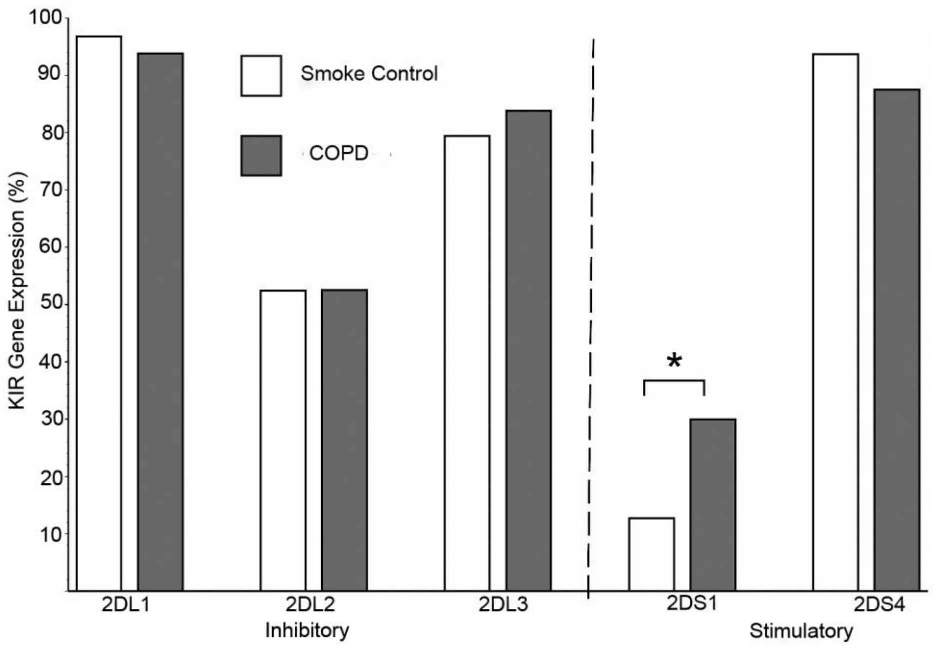

An initial pilot trial determined KIR prevalence in 80 COPD and 63 Smoke Control (SC) subjects. Intergroup differences were most evident in KIR2DS1. Subsequent analyses of subjects focused on this gene. Comparisons made by Chi-square (*p = 0.014).

Online Supplement Figure 2.

Separation of Peripheral Blood Mononuclear Cells (PBMNC) into NK Cell Depleted and NK Enriched Subpopulations

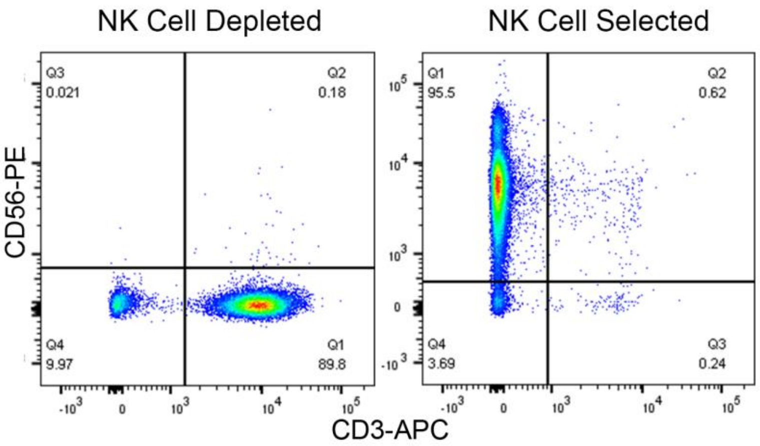

Flow cytometry was used to confirm the purity of PBMNC depleted of NK cells ($CD56^+$) by fluorescence activated cell sorting (left panel) and of NK cell enrichments by negative selections of other leukocytes (described in text) (right panel). The majority of the $CD56^{null}$ cells among the PBMNC were $CD3^+$ lymphocytes (T-cells).

Online Supplement Figure 3.

Verification of HLA-C allele transfection into K562 cells

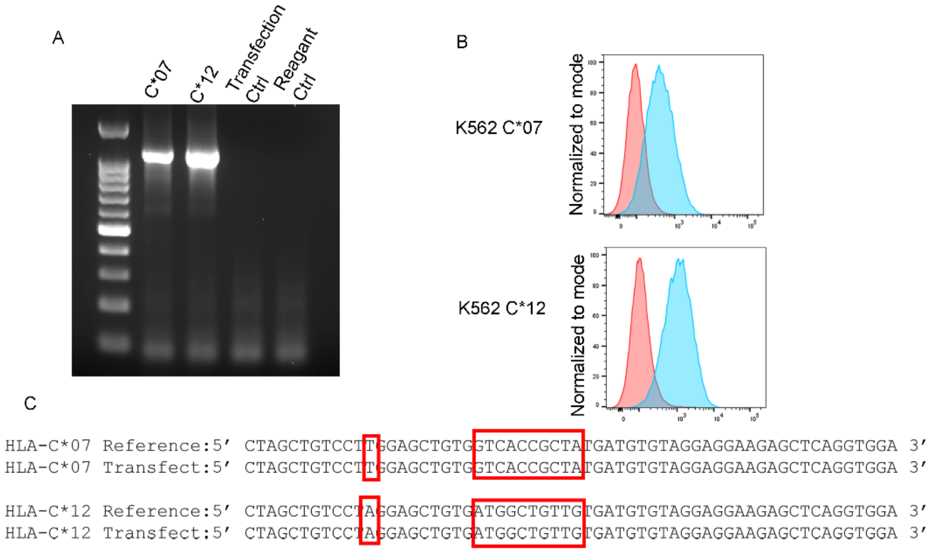

Verification of HLA-C allele transfections in K562 cells. **A**. Agarose gel electrophoresis of PCR product showing successful transfection of 1101bp sequence HLA-C alleles (Lanes from Left to right) 1. DNA Ladder (100bp); 2. K562 transfected with HLA-C*07; 3. K562 transfected with HLA-C*12; 4. K562 controls transfected with empty vector (Transfection ctrl); 5. Reagent Control (Reagent ctrl). **B.** Flow cytometry showing expression of HLA-C*07 and HLA-C*12 in transfected K562 cells. *Red= Control cells transfected with empty vector and *Blue= Cells transfected with HLA-C. **C.** DNA nucleotide sequence in region of greatest dissimilarity between HLA-C*07 and HLA-C*12 (961bp to 1020bp) showing the published reference sequence and sequences in the HLA-C-transfected K562 cells.

References:

1. Robinson J, et al. IMGT/HLA database--a sequence database for the human major histocompatibility complex. *Tissue Antigens* 2000; 55: 280-287.
